# Cold Stress Responses and Adaptation Mechanisms in *Moringa oleifera* Lam.: A Metabolite-Centred Review

**DOI:** 10.3390/plants15060960

**Published:** 2026-03-20

**Authors:** Blair Moses Kamanga, Donita L. Cartmill, Craig McGill, Andrea Clavijo McCormick

**Affiliations:** School of Agriculture and Environment, Massey University, Tennent Drive, Palmerston North 4410, New Zealand; b.m.kamanga@massey.ac.nz (B.M.K.); d.cartmill@massey.ac.nz (D.L.C.); c.r.mcgill@massey.ac.nz (C.M.)

**Keywords:** antioxidative defence, cold stress, hormonal regulation, metabolites, morphological traits

## Abstract

*Moringa oleifera* Lam. (moringa) is a desirable crop for intensive cultivation because of its multiple uses in human and animal nutrition, medicine, and ecological applications. Its resilience and adaptability to various environmental conditions make it an attractive option for farmers seeking alternative cash crops that can thrive in challenging agricultural environments. While its resilience is well documented in tropical and subtropical climates, limited information exists on its growth dynamics and adaptation mechanisms to prolonged cold stress, which constrains its expansion and cultivation in temperate regions. This review synthesises current knowledge on cold stress adaptation mechanisms and the coordinated functional roles of primary and secondary metabolites in response to cold stress in plants, with a focus on moringa. Although considerable progress has been made in understanding morphological adjustments to cold stress in moringa plants, limited attention has been given to elucidating the physiological, metabolic, and genetic regulatory mechanisms underlying its cold-adaptive responses. Moreover, despite the potential roles of primary and secondary metabolites in coordinating protective functions against cold stress in plants, specific metabolites and their functional roles against cold stress remain insufficiently characterised in moringa. While genetic improvement and selective breeding have improved key agronomic traits, including growth rate, biomass yield, and nutritive value, breeding for enhanced cold stress tolerance remains insufficiently explored. Future studies should focus on integrative metabolite profiling, as well as the identification and selection of cold-tolerant provenances, to support the development of cold-tolerant gene pools to expand the cultivation range of moringa into temperate regions.

## 1. Introduction

Low temperature is a critical abiotic stress factor that compromises plants’ growth and limits their geographical distribution [1,2]. Exposure to low temperature induces morphological, physiological, molecular, biochemical, and hormonal alterations that disrupt normal plant function, growth and productivity [3,4,5]. Cold stress injury (chilling and freezing) not only causes a reduction in crop productivity and quality in cultivated crops but also affects morphogenesis and may induce cold acclimation [6,7]. Cold acclimation refers to an increased tolerance to freezing that develops through repeated exposure to low, non-freezing temperatures and involves coordinated physiological, morphological, biochemical, and cellular reconfigurations [8,9,10].

While plants from tropical and subtropical environments are typically sensitive to cold stress and lack cold acclimation capacity, temperate species are tolerant to seasonal climatic fluctuations and easily adapt to cold stress [8,9,10]. Cold adaptation mechanisms can be broadly classified as either avoidance (i.e., transpiration cooling, stomatal closure, leaf orientation, and early maturation) or tolerance (i.e., altered transcripts, free radical scavengers, and the production of osmo-protectants) [11].

*Moringa oleifera* Lam. (moringa) is one of the most cultivated and versatile tropical plants and is adapted to a wide range of environments [12]. Moringa is highly valued for its nutritional and medicinal uses for humans, and its leaves, flowers, seeds, and roots are utilised in agriculture for livestock feed and in natural resource management [13]. Its resilience and adaptability to various environmental conditions make it an attractive option for farmers seeking alternative cash crops in different agricultural systems including areas outside its natural range [14]. 

The species’ worldwide distribution and adaptation to various agroecological zones could be attributed to a wide range of genetic variation and adaptation mechanisms among cultivated and wild provenances [15,16]. Moringa also produces a variety of secondary metabolites with biological activity linked to competitive advantage, stress responses, and defence, allowing the plant to thrive in new environments [14]. However, the sensitivity of moringa to cold stress poses a major natural barrier for its natural expansion and introduction into temperate regions [17]. Hence, this review explores the growth dynamics and adaptative mechanisms of moringa in response to cold stress by integrating research findings from recent studies. It further proposes avenues for future research that can be integrated into the development of cold-tolerant cultivars suitable for temperate environments.

## 2. Literature Rationale and Review Methodology

Although considerable progress has been made in characterising the morphological adjustments of moringa to low temperatures [4,5], less effort has been directed towards the mechanisms underlying metabolic, physiological, biochemical and genetic regulation. This knowledge gap constrains a holistic understanding of integrative and coordinated strategies that enable moringa to perceive, respond to, and tolerate cold stress. Therefore, multidisciplinary studies to bridge morphological, physiological, and biochemical regulations are essential to address the following research questions: what are the growth dynamics of moringa and its adaptation mechanisms to cold stress? What information is needed for effective breeding programs for cool-tolerant cultivars? Does moringa possess coordinated primary and secondary metabolites that are responsible for cold stress adaptation?

To address the above questions, a systematic review was conducted to synthesise recent advances in growth dynamics, adaptative mechanisms, and breeding initiatives associated with moringa. As a complementary line of inquiry, the coordinated roles of primary and secondary metabolites in the plant cold stress response were examined to clarify how metabolically driven adjustments may contribute to the broad ecological distribution of this species. The comprehensive search for relevant scientific literature on moringa across various scientific databases, e.g., Google Scholar, Web of Science, PubMed, and Scopus, was conducted using a combination of keywords, including ‘moringa’ AND ‘adaptation mechanisms’, ‘moringa’ AND ‘growth dynamics’, ‘moringa’ AND ‘breeding’, ‘moringa’ AND ‘genetics’, to obtain in-depth information on the current knowledge of various adaptation mechanisms and growth dynamics. Article suitability was determined by evaluating methods aimed at screening titles and abstracts, which have been described as an efficient approach for identifying relevant literature on a specific topic of study [18].

## 3. Adaptation and Potential Distribution of Moringa in Temperate Regions

Conflicting reports exist regarding the growth dynamics and climate suitability of moringa, particularly in relation to temperature and precipitation gradients. Earlier studies characterised moringa as adapted to arid and semiarid tropical environments [17], with optimal growth observed between mean temperatures of 12 °C and 40 °C [19]. However, emerging evidence indicates a broader thermal tolerance than previously reported. Recent studies have demonstrated that moringa can withstand light frost to −3 °C, whereas some provenances exhibit a freezing tolerance threshold (LT_50_) of approximately −2.8 °C [4].

Conversely, moringa is sensitive to severe or prolonged freezing, with significant tissue injury reported at temperatures below −5 °C [20]. Supporting evidence indicates that moringa may survive transient frosts between −1 °C and −3 °C during cooler months but can also endure high thermal extremes of up to 48 °C under arid conditions [13]. These contrasting findings emphasise the substantial degrees of ecotypic variation and physiological plasticity within the species. Nevertheless, despite its broad adaptive range, moringa achieves optimal growth, photosynthetic performance, and biomass accumulation with a moderate thermal range of 25–35 °C [12]. However, laboratory experiments have demonstrated the importance of exposure duration; for example, a 10/5 °C Day–night temperature regime sustained for 8 days resulted in unrecoverable injury after a 4-day recovery period [4].

Previous studies provide a quantitative threshold for freezing injury but should be interpreted cautiously since tolerance varies with growth stage and experimental conditions [21]. For example, seedlings are more frost-sensitive whereas established plants may tolerate short temperature declines due to greater structural protection and carbohydrate reserves [22]. Cellular damage in moringa under freezing conditions could be due to the extracellular ice formation and cellular dehydration, which disrupt membrane integrity and metabolic activity as shown for other tropical species such as oil palm (*Elaeis guineensis* Jacq.) [23], nevertheless, recent studies suggest that moringa shows little or no capacity for cold acclimation [4].

In New Zealand, studies on climate suitability have identified the top of the North Island, i.e., Northland, and the surrounding microclimates as suitable for the introduction and cultivation of moringa [24]. These locations are characterised by a mean annual temperature of approximately 25 °C, with annual precipitation ranging from 2000 mm to 2700 mm [25]. These conditions align closely with the optimal thermal and hydric requirements previously reported for moringa establishment and cultivation, where temperatures ≥ 25 °C and rainfall exceeding 1000 mm promote vigorous growth and sustained physiological performance [12,26]. This climatic congruence between these North Island zones and moringa’s native or naturalised habitats demonstrates the species’ potential adaptability to subtropical environments.

## 4. Adaptation Mechanisms of Moringa to Cold Stress

**Figure 1:**
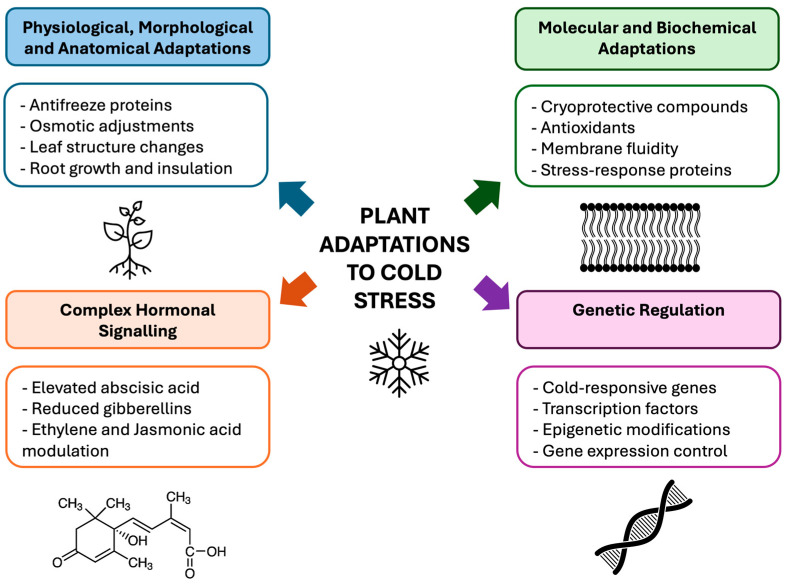
Integrated mechanisms underlying cold adaptation in plants. The diagram illustrates the multi-layered responses plants employ to perceive and withstand low-temperature stress. Physiological, morphological, and anatomical changes including antifreeze protein accumulation, osmotic adjustment through compatible solutes, alterations in leaf structure, and root system insulation help to maintain cellular stability during freezing conditions. Molecular and biochemical adaptations involve the production of cryoprotective metabolites, activation of antioxidant systems, adjustments in membrane lipid composition to preserve fluidity, and induction of stress-responsive proteins such as dehydrins. Cold-induced shifts in hormonal balance, i.e., elevated abscisic acid (ABA), reduced gibberellins (GA), and modulatory roles of ethylene and jasmonic acid (JA), coordinate growth restraint with activation of defense pathways. These processes converge under genetic regulation which is mediated by calcium-dependent signaling cascades, transcription factors, cold-responsive (COR) gene expression, and epigenetic modification that improves transcriptional responses to cold stress.

As sessile organisms unable to escape adverse conditions, plants depend on a suite of integrated adaptation mechanisms to withstand harsh environmental conditions including cold stress. These mechanisms encompass coordinated physiological, morphological, anatomical, molecular, biochemical, hormonal, and genetic responses that enhance plant tolerance to low temperatures (Figure 1) [27,28,29,30,31,32,33,34]. Physiologically, plants mitigate freeze-induced cellular damage through antifreeze proteins, osmotic adjustment through compatible solute accumulation, and structural modifications such as thicker cuticles, altered leaf morphology, and insulated root systems [5,35,36]. At molecular and biochemical level, cold stress triggers the synthesis of cryoprotective metabolites, activation of antioxidant defences, remodelling membrane lipid composition to maintain fluidity, and induction of stress-responsive proteins including dehydrins and late embryogenesis abundant (LEA) proteins [29,35,37,38]. These processes are strongly integrated with shifts in hormonal homeostasis, where increased abscisic acid (ABA) promotes cold-responsive gene expression and stomatal regulation, reduced gibberellin (GA) levels suppress growth to conserve energy, and ethylene and jasmonic acid (JA) modulate cross-talk among stress pathways [39]. Ultimately, cold acclimation is governed by complex genetic regulation involving calcium-mediated signalling cascades, activation of C-repeat/dehydration-responsive element binding factors (CBF/DREB), expression of cold-responsive (COR) genes, and the epigenetic modifications that adjust chromatin structure to improve stress-responsive transcriptional processes [7,29,40,41,42,43].

### 4.1. Physiological, Morphological, and Anatomical Mechanisms in Moringa

Plants possess various morphological adaptation mechanisms in response to cold stress through phenotypic plasticity, such as reduced leaf area, thicker cuticles, and increased root-to-shoot ratios [44]. These mechanisms minimise energy loss, mitigate freezing damage, and maintain productivity. Stomatal modification is an anatomical defence mechanism against cold stress aimed at promoting water use efficiency and stress tolerance [45,46]. In moringa, the number of stomata per unit leaf area was significantly greater at lower temperatures (10/20 °C; day/night temperatures) than at 20/30 °C; although the stomata were larger at higher temperatures. The lower stomatal density at 20/30 °C resulted in a reduction in leaf conductance [5]. The decline in leaf conductance limits the diffusion of CO_2_ into the leaf mesophyll for photosynthesis, reduces the catalytic activity of ribulose-1,5-bisphosphate carboxylase/oxygenase (RuBisCO), and consequently compromises plant growth and productivity [47].

Leaves of moringa plants grown under a low-temperature regime of 10/20 °C and a 10 °C decrease in day and night temperature presented an average increase in leaf thickness of 43.1%, i.e., from 0.14 mm to 0.24 mm [5]. Plants grown at 10/20 °C also presented more spongy mesophyll tissue and longer palisade cells than did the leaves of plants grown at 20/30 °C [5]. Plants modify their leaf structure in response to cold stress to stimulate tolerance. They exhibit specific morphological and anatomical adaptations, such as increased palisade thickness, increased tightness of the tissue structure, and an increased palisade spongy mesophyll tissue ratio, to minimise the effect of chilling stress [48,49] Similar trends have been observed in other plants, e.g., Mastic tree (*Pistacia lentiscus* L.) [50] and Shearer’s Phoebe (*Phoebe sheareri* Hemsl.) [51]. Modifications in leaf anatomy across plant species provide evidence of how plants adapt to and thrive in temperate regions.

The thickening of leaves observed in moringa serves as a protective mechanism to minimise water loss and maintain cellular integrity under cold conditions. For example, exposing moringa plants to 15/10 °C for 96 h increased leaf thickness by 17.3%, with elongated and swollen spongy mesophyll cells [3], while the increase in palisade thickness suggests that plants allocate more resources to photosynthetic tissue in response to chilling stress. Plants usually increase the efficiency of photosynthesis by minimising the damaging effects of photoinhibition at lower temperatures [52].

Cold stress compromises the integrity of the thylakoid membrane in tropical plant species, which is the primary site of electron transport and photochemical reactions. This results in reduced membrane fluidity, impaired photosynthetic efficiency, and increased electrolyte leakage due to oxidative stress [53]. Moreover, cold-stressed plants exhibit a reduction in the contents of chlorophyll a and b, which are key pigments essential for light absorption and energy conversion during photosynthesis. This reduction is indicative of broader disruption of photosynthetic function and overall physiological impairment [8,54]. In moringa plants, cold stress at 15/10 °C and 10/5 °C decreased the maximum quantum yield of photosystem II (F_v_/F_m_) by 28.7% and 73.7%, respectively, with full recovery after one day of exposure under both temperature regimes [4]. Nevertheless, prolonged exposure of the plants for four days exacerbated the effect, with reductions of 32.2% and 87.7% at 15/10 °C and 10/5 °C, respectively, without recovery. The reduction in F_v_/F_m_ at 10/5 °C may indicate the failure of moringa provenances to adapt to or defend against cold stress over long exposure periods.

### 4.2. Molecular and Biochemical Adaptation Mechanisms in Moringa

Cold stress influences the production and accumulation of primary and secondary metabolites to counteract the effects of cold stress, which disrupts biochemical and hormonal processes in plants [52]. Research has shown that plants respond differently to cold stress on the basis of species type and stress-dependent effects. For example, exposing moringa plants to 15/10 °C for 48 and 96 h resulted in increases in phenolic contents of 21.6% and 26.5%, respectively [3]. Similarly, in grapes (*Vitis vinifera* L.), more tolerant cultivars were characterised by higher contents of phenolic compounds and better radical-scavenging capacity [55]. This type of response is indicative of an adaptation strategy, as phenolic compounds modulate the activity and expression of key antioxidant enzymes to increase the cellular redox balance under cold stress [56]. Nevertheless, specific metabolites responsible for cold acclimation and tolerance in moringa have yet to be characterised.

The accumulation of non-structural carbohydrates, phenolics, and proteins in plants has been previously reported as a response mechanism to cold stress. For example, in *Jatropha curcas* L., the accumulation of soluble sugars, including starch, maltose, sucrose, glucose, galactinol, and raffinose, was observed at 12 °C (6–48 h) [34]. These sugars function not only as osmoprotectants and energy reserves but also as signalling molecules that regulate stress-responsive gene expression to increase membrane stability and mitigate oxidative damage [57]. Cryoprotective sugar alcohols, including sorbitol, ribitol, and inositol, are known to accumulate under cold stress conditions; these polyols protect cellular structures through maintaining membrane fluidity, scavenging ROS, and reducing the risk of freeze-induced injury in plant leaves [58].

Contrasting patterns have been observed regarding proline and carbohydrate accumulation under elevated temperature conditions [59], with earlier findings indicating an increase in phenolic contents with decreasing temperature [3]. Several studies have shown that exposure to abiotic stresses such as cold induces the accumulation of polyamines such as spermidine, spermine, and putrescine in *Brassica napus* L. [60]. However, there is insufficient information on the changes in polyamines in moringa plants that survive light frost. In general, the total soluble sugar, free amino acid, proline, phenolic, and flavonoid contents of leaves increase when moringa plants are exposed to abiotic stress conditions as survival mechanisms [59].

### 4.3. Complex Hormonal Balance as an Adaptation Mechanism in Moringa

Most plants, including moringa, contain numerous phytohormones, such as abscisic acid (ABA), auxins, cytokinins, and gibberellins [29,61,62]. As a key stress-responsive phytohormone in plants, ABA coordinates complex regulatory networks that enable plants to perceive and adapt to cold-induced cellular dehydration and oxidative stress [63]. During cold stress, ABA functions as a regulatory hormone to increase cold tolerance through the regulation of stress-responsive genes, the accumulation of protective metabolites, and increased membrane integrity [62,63,64].

Elevated ABA levels activate the expression of cold-responsive (COR) and late embryogenesis abundant (LEA) genes to produce antifreeze proteins and osmoprotectants that stabilise cellular structures and cold-induced dehydration [37,65]. For example, studies in common beech (*Fagus sylvatica* L.) have shown that ABA mediates stomatal closure to reduce cuticular transpirational water loss and stimulates antioxidant defense systems that mitigate oxidative damage [63,66]. However, the level of ABA in moringa provenances that survive brief periods of chilling and light frost stress down to −2 °C [4] has not been investigated to establish its adaptive course. Therefore, further studies are needed to establish the role of ABA and other phytohormones in moringa in relation to cold tolerance.

Plant hormones such as ABA, auxins, cytokinins, and ethylene play important roles in regulating the expression of NAC transcription factors [*No Apical Meristem (NAM); Arabidopsis transcription activation factor (ATAF1/2); and cup-shaped cotyledon (CUC2)*] [40]. These transcription factors directly or indirectly regulate plant gene expression in response to developmental and environmental stress [40]. Both ABA and NAC transcription factors are widespread in plants [67], suggesting these may play a role in moringa responses to cold stress.

### 4.4. Genetic Regulation as an Adaptation Mechanism in Moringa

Plants synthesise and produce large numbers of microRNAs (miRNAs) known to control a variety of plant functions in response to biotic and abiotic stressors [68]. These noncoding RNAs regulate the expression of genes in eukaryotic cells (21–24 nucleotides), are transcribed as primordial miRNAs, and are precursors of RNAs by RNA polymerase-II inside the nucleus [69]. Research also suggests that plants subjected to different environmental stress factors, including cold, synthesise stress-responsive proteins to increase their survival under adverse conditions [7,40]. The induction of miR171d and miR2118a in moringa plants exposed to cold stress in dark conditions suggests a genetic component of the cold stress adaptation mechanism since these miRNAs are known to regulate plant gene expression in response to various abiotic stimuli [7,70,71].

Furthermore, the elevated expression levels of specific miRNAs in moringa leaves and callus tissues exposed to cold stress suggest that the activation of post-transcriptional regulatory pathways enhances stress tolerance [72]. The ability of moringa to thrive and adapt across diverse climatic environments demonstrates its underlying genetic variability, which may account for the differential adaptive mechanisms observed among the provenances and their associated effects on relative growth rates under varying environmental conditions [73,74].

Most plants survive and reproduce under adverse temperatures because NAC factors are among the adaptation processes. They play essential roles in various stress responses, including low temperature, drought, and pathogen attack [75]. These gene families form a complex regulatory network that modulates the expression of stress-responsive genes, including those involved in detoxification, osmotic regulation, and antioxidant defense, to optimise plants’ chances of survival under stress conditions [75]. For example, gene expression (NAC) was evident in *Madhuca longifolia* (L.) J.F.Macbr. plants [76] and alfalfa (*Medicago sativa* L.) [77] under cold stress. 

Meta-analyses have demonstrated that overexpression of NAC transcription factors, i.e., PbeNAC1 (*Pyrus betulifolia* NAC1) and SlNAC (*Solanum lycopersicum* NAC), enhances cold tolerance by activating cold-responsive regulatory pathways that lead to improved cell membrane stability and increased scavenging of ROS under low-temperature stress [78,79]. The ability of certain moringa provenances to survive cold stress provides evidence of the existence of genotypes with desirable traits that could be enhanced through breeding and utilised for cultivation in temperate regions.

## 5. Coordinated Functional Roles of Metabolites in Response to Cold Stress

**Figure 2:**
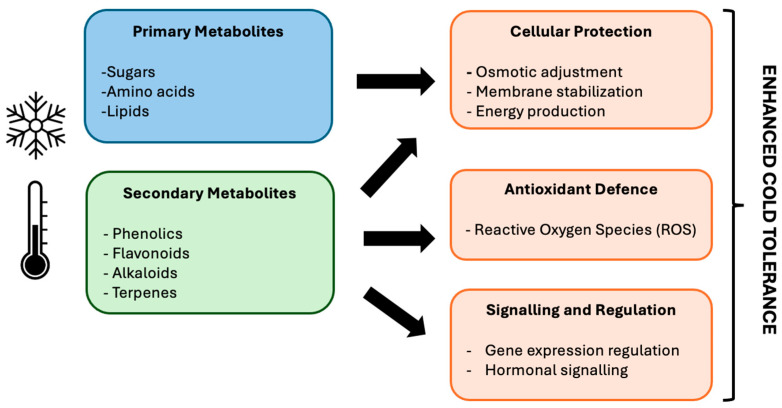
Schematic illustration of coordinated roles of primary and secondary metabolites in plant cold stress responses. Primary metabolites support osmotic adjustment, membrane stabilisation, and energy balance, while secondary metabolites contribute to antioxidant defence and signalling functions to enhance cold tolerance in most plant species.

Plants native to arid and semiarid tropical environments are susceptible to cold stress (chilling at 0–15 °C or freezing at <0 °C). Cold stress causes membrane rigidification in plant cells, which has been described as a primary biophysical alteration that triggers downstream signalling cascades [1]. Initially, there is a transition phase that compromises membrane fluidity and affects embedded proteins; this disrupts enzymes and leads to photoinhibition, membrane damage, and reduced photosynthetic capacity in most plant species [80]. At the molecular level, chilling stress interferes with gene expression and translation by promoting the formation of RNA secondary structures [29]. When freezing stress is more severe, it can cause lethal and physical damage to plant organs and tissues. Freezing injury begins with the nucleation of extracellular ice, which induces cellular dehydration and mechanical injury, resulting in plant death [81]. Metabolic shifts in response to environmental stressors represent strategic physiological, biochemical, and hormonal reconstitutions aimed at increasing the survival rate and productivity. As sessile organisms, plants deploy various adaptation mechanisms associated with complex interactions between primary and secondary metabolism [82]. Figure 2 illustrates the coordinated roles of primary and secondary metabolite-driven pathways culminating in enhanced cold tolerance in plants. These processes could be linked and contribute to the adaptive responses of moringa plants to environmental stressors including low-temperature stress by supporting cellular protection and physiological stability.

### 5.1. Coordinated Functional Roles of Primary Metabolites in the Response to Cold Stress

The accumulation of soluble sugars is one of the adaptation mechanisms that plants use to survive cold stress [8]. Low temperatures induce rapid accumulation of soluble carbohydrates, including glucose, sucrose, fructose, and maltose, which are all essential primary metabolites [83]. They perform multiple functions as compatible osmolytes, cryoprotectants, scavengers of ROS, and signalling molecules [64,84]. These sugars not only function as osmolytes but also stabilise membranes and proteins to protect cellular structures during freeze–thaw cycles. They provide the energy required for various metabolic functions, such as respiration, photosynthesis, and the transportation of molecules, under extremely cold conditions [53].

The accumulation of soluble carbohydrates such as disaccharides, e.g., trehalose and sucrose; oligosaccharides such as raffinose and stachyose; and polymers of fructose molecules, has been linked to increased freezing tolerance in various temperate species such as *Jatropha curcas* L. seedlings [34]. These carbohydrates, together with their corresponding metabolic enzymes, play essential roles as compatible osmolytes and scavengers, enabling plants to maximise their productivity under cold stress [54,84].

Cold stress delays plant growth due to cell death or cellular injury; this decreases membrane integrity, causes malfunctions in antioxidant defences and enzyme activities, and modulates hormonal signalling responses [29,36]. This results in the low production of energy required for plant growth and development. However, primary metabolites such as proline and γ-aminobutyric acid (GABA) accumulate during cold stress conditions to help with ROS detoxification, redox buffering, and osmotic adjustment [85,86]. Moreover, GABA accumulates to provide an alternative pathway for energy production and the modulation of intercellular pH and signalling [87]. In other plants, cold stress has been shown to induce the accumulation of GABA and proline. For instance, in *Medicago ruthenica* (L.) Trautv. [88] and tea plants (*Camellia sinensis* L.) [89], these metabolites contribute to osmotic regulation, stabilisation of cellular structures, and mitigation of oxidative damage [85,89]. Although such responses have not been extensively characterised in moringa, similar metabolic adjustments may contribute to its physiological response to cold stress and play a significant role in maintaining cellular homeostasis under adverse environmental conditions. These findings further suggest that moringa plants maintain cellular homeostasis during cold stress due to increased synthesis of primary metabolites, indicating a conserved energy strategy for adaptation.

### 5.2. Coordinated Functional Roles of Secondary Metabolites in the Response to Cold Stress

While primary metabolites are involved in plant growth activities, secondary metabolites are synthesised as defense and plant communication mechanisms under various stressors [90]. Secondary metabolites such as flavonoids, alkaloids, and terpenoids are regulated under cold stress, function as antioxidants and play regulatory roles in adaptation mechanisms [30,64]. This type of adaptation mechanism involves the synthesis and production of complex chemical compounds that contribute to the structural function of various metabolic processes for plant survival and productivity.

The cold-induced accumulation of phenolic acids (gallic, caffeic, protocatechuic, and chlorogenic acids) in plant cells decreases the freezing point, maintains water potential, and protects the cells from bursting [91]. The deposition of phenolic compounds into the cell wall matrix through increased lignification due to cold stress has been thoroughly reviewed [92]. In moringa, cold stress may stimulate the accumulation of phenolic compounds and increased lignification within the cell wall matrix, which can contribute to structural reinforcement and protection against cellular damage. The subsequent thickening of the cell wall strengthens its mechanical properties to mitigate freeze-induced dehydration and cellular distortion. This structural reinforcement is a critical component of the overall freezing tolerance mechanism of plants. Additionally, flavonoids such as quercetin, kaempferol, and anthocyanins mitigate photoinhibition by filtering excess light and harmful ROS to protect photosystem integrity through cold-induced stomatal closure under altered light conditions [93].

The dynamic adjustment of terpenoid metabolism and accumulation in plants is due to its role in cold stress tolerance through various protective mechanisms, such as membrane stabilisation, antioxidant activity, and hormonal signalling [42]. However, the mechanisms behind this accumulation still lack a systematic explanation. Hence, understanding cold response mechanisms in plants remains complex and ambiguous. Nonetheless, carotenoids, monoterpenoids, and sesquiterpenes function as antioxidants and cell membrane stabilisers in most plants, whereas some terpenoids play crucial signalling roles during cold acclimation [94]. Terpenoids further play a vital role in lipid metabolism and accumulation. For example, the exposure of walnut (*Juglans regia* L.) plants to cold stress increased the concentration of 3,7,11,15-tetramethyl-2-hexadecen-1-ol (Phytol) to mitigate the effects of cold injuries [95].

Research has shown that plants increase the synthesis and production of glucosinolates (GSLs) in response to cold stress as an adaptation mechanism [30,96]. GSLs are a class of specialised metabolites found abundantly in the brassica family, where they contribute significantly to cold stress tolerance by modulating key physiological processes and interacting with other defence pathways [97]. Cold stress alters the balance of GSL types, which in turn affects the overall concentration and expression of genes involved in their synthesis [98]. Under normal conditions, GSLs tend to be inactive, but they are hydrolysed upon tissue damage due to cold stress caused by the enzyme myrosinase [97]. The hydrolysis of GSLs results in the production of chemical compounds with antioxidant properties that help scavenge the ROS generated during cold stress. However, the mechanisms underlying the accumulation of GSLs in moringa plants in response to cold stress have not been thoroughly studied to understand their specific roles in adaptation mechanisms.

There is a clear interaction between primary and secondary metabolism in adaptation mechanisms to cold stress. For example, carbohydrates are produced and accumulate during primary metabolism and serve as precursors for the synthesis of secondary metabolites to prevent freezing stress [99]. While sugar-derived precursors are fed into the phenylpropanoid and terpenoid pathways, amino acid metabolism coincides with polyamine and alkaloid biosynthesis to counteract the effects of cold stress [85,100]. This phenomenon not only helps plants during stress acclimation but also helps them optimise growth and defense mechanisms under suboptimal temperatures. The relationship between primary and secondary metabolites in connection with cold stress indicates high metabolic coordination in response to environmental stressors. However, this type of coordination has yet to be characterised in moringa, especially for provenances that have shown brief tolerance to cold.

## 6. Research Progress on Genetic and Breeding Programs for Cold Tolerance in Moringa

The genetic patterns and morphological traits of moringa have been studied to select provenances with desirable agricultural traits [101]. Since 1976, the Indian Council of Agricultural Research-National Bureau of Plant Genetic Resources (ICAR-NBPGR) has been conducting extensive exploration and germplasm collection of selected vegetable crops, including moringa, for improvement of agronomic traits [102]. Global efforts to collect, select, conserve, and exchange moringa germplasm have been a driving force for the identification of elite provenances for breeding programs of new cultivars with yield-related traits [61].

Additionally, MC Palada [103] reported that the World Vegetable Centre has distributed moringa germplasm to research institutions, non-governmental organisations, and private companies in more than 15 countries to promote moringa production. These programs have transformed and contributed to the genetic improvement of major quantitative and qualitative traits of moringa [104]. However, current breeding programs have focused more on yield and its associated quality parameters.

The current genetic diversity assessment of moringa has provided an opportunity to develop various cultivars, including two novel cultivars with high-yielding traits (PKM-1 & PKM-2), for commercial cultivation [61]. Although moringa is regarded as a “miracle tree” with numerous benefits, the plant remains neglected in breeding for cold tolerance [105]. Targeted improvement of this trait is essential for supporting species expansion, resilience, and reliable production in temperate environments.

Induced mutagenesis via a low dose of ethyl methane sulfonate (EMS) produced enhanced leaf mutant lines with potential germplasm and functional genomic resources for improving leaf architecture, nutrient yield, and adaptive traits to stressors [106]. While new cultivars have been developed, molecular breeding efforts in moringa remain limited compared with those in other major crops [107]. For example, advancements in molecular marker development for germplasm characterisation and the application of DNA barcoding in moringa remain minimal [108,109]. However, the availability of genomic resources in moringa, such as SSR markers, genome sequence data, and other biotechnological tools [110,111], provides a foundation for breeding new cultivars with desirable traits suitable for cultivation in temperate regions. 

## 7. The Current Knowledge Gap and Potential Research Perspective of Moringa

Despite moringa exhibiting resilience across a range of abiotic stresses, the mechanistic basis behind this adaptability remains insufficiently characterised. Emerging evidence shows that in most plants, adaptive capacity is mediated through complex reconfigurations of primary and secondary metabolic pathways. These processes enable dynamic adjustments in osmotic balance, energy allocation, and redox homeostasis for plant survival in stressful environments [8,40,65,112,113]. While most of these mechanisms have been extensively studied in other plants through metabolomics analyses, coordinated regulation between metabolic networks and their integration with transcriptional, hormonal, and signalling cascades in moringa remain insufficiently studied. This information, alongside other resources, is critical to inform successful modern cold-breeding programmes in moringa.

Metabolomics (i.e., the systematic study of the unique chemical fingerprints left behind by plant metabolic processes under specific conditions) offers a powerful framework for advancing biochemical research aimed at breeding cold-tolerant crop varieties [114,115]. By profiling metabolites such as soluble sugars, proline, polyamines, and antioxidants under chilling and freezing conditions, metabolomic analyses allow the identification of biochemical markers that consistently distinguish tolerant from sensitive genotypes and thus can be used as robust selection tools in breeding programmes [116]. Beyond marker discovery, metabolomics also provides mechanistic insights by mapping coordinated changes in primary and secondary metabolic pathways during cold stress, thereby linking observed physiological traits, such as membrane stabilization, reactive oxygen species (ROS) detoxification, and improved osmotic adjustment, to their underlying biochemical networks and regulatory circuits [35].

Comparative metabolite profiling further enables early discrimination between cold-tolerant and cold-sensitive genotypes at seedling or early vegetative stages, significantly accelerating selection compared with reliance on later field-based winter survival data. When integrated with genetic data through QTL mapping, GWAS, or metabolite-QTL analyses, metabolomics strengthens candidate gene discovery by establishing direct links between metabolite signatures and genomic regions controlling cold-tolerance traits [116,117], thereby enhancing the precision of marker-assisted and genomic selection strategies. Finally, metabolomic studies that track metabolic reprogramming during cold acclimation, de-acclimation, and re-acclimation provide crucial insights into genotype resilience under increasingly variable winter temperature regimes, informing breeding decisions for climate-adapted cultivars capable of maintaining performance under future environmental fluctuations [116,117].

**Figure 3:**
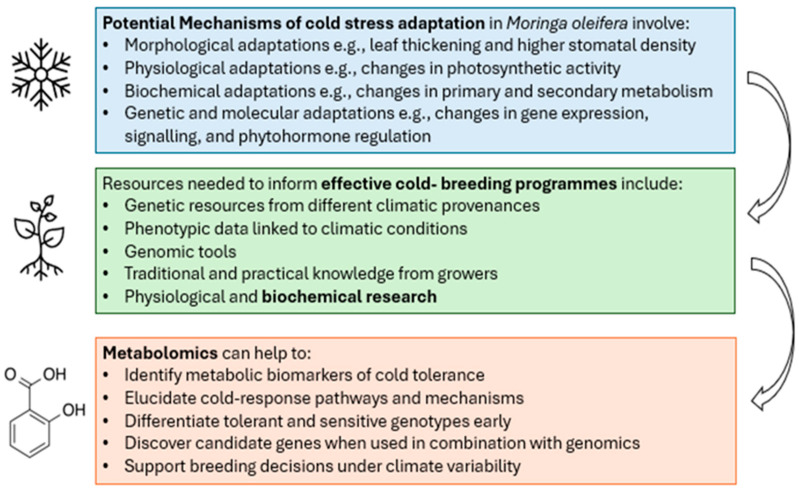
Considerations for breeding of cold-tolerant varieties of moringa, and the role of metabolomics in contributing to biochemical research.

In Figure 3, we provide a schematic summary of potential mechanisms of cold stress adaptation in moringa, the resources needed to develop effective cold-breeding programmes and how metabolomics can contribute to these efforts.

Future studies should prioritise integrative approaches that combine metabolomic profiling with genomic, transcriptomic and epigenomic assessments to identify metabolites, genes and regulatory networks associated with cold tolerance in moringa. Additionally, biochemical and molecular approaches linking carbon and nitrogen metabolism with the biosynthesis of secondary metabolites such as phenolics, glucosinolates, and flavonoids are strongly encouraged. Moreover, most studies have been confined to foliar tissues, neglecting the metabolic versality of other plant organs. Roots comprise a significant portion of plant biomass and are essential to water and nutrient absorption, as well as to ecological interactions, making their study a fundamental piece of the puzzle. Strategic provenance selection from cold environments coupled with omics-driven breeding programs is vital for new cultivars suitable for temperate regions.

Table 1 highlights some of the current knowledge, research gaps, and future prospects that can help in understanding multiomics integration and functional validation to unravel the metabolic networks governing stress resistance in moringa. A comprehensive understanding of these processes not only defines or clarifies the adaptive plasticity of a species but also provides a biochemical framework for enhancing stress tolerance through metabolic engineering and sustainable crop management.

## 8. Conclusions

Moringa is a promising multipurpose crop with high nutritional, medicinal, and ecological value. While the plant is resilient under tropical and subtropical climates, its cultivation in temperate climates is constrained by its sensitivity to low temperatures. There is limited information on moringa growth dynamics under cold stress. However, the available evidence indicates that moringa deploys a suite of morphological adjustments, physiological acclimatisation, biochemical modulation, and genetic regulation as cold adaptation mechanisms. The coordinated functional roles of primary and secondary metabolites play a central role in stress mitigation in plants; however, their precise roles and regulatory pathways in moringa remain insufficiently understood. The establishment of a joint network among research institutions working on moringa, and engagement with current moringa growers and traditional knowledge owners, would generate and provide important information vital for the development of cold-tolerant germplasms and production systems in temperate regions. Metabolomics and its integration with other -omics and information sources promises to be a helpful tool in advancing plant breeding to develop cold-tolerant varieties.

## Figures and Tables

**Table 1:** Research gaps and future directions for the coordinated regulation of primary and secondary metabolites in moringa under abiotic conditions.

Aspect	Current Understanding	Knowledge Gap	Future Research Direction
Metabolomic coordination under stress	Both primary (sugars, amino acids, organic acids) and secondary metabolites (e.g., phenolics, flavonoids, glucosinolate) contribute to stress mitigation in plants [58,118]	The integrative coordination between primary and secondary metabolic networks under specific abiotic stress conditions (e.g., cold) remains poorly defined in moringa.	Use integrative metabolomic–transcriptomic approach to define network-level coordination and cross-regulation of metabolic pathways under cold stress.
Regulatory/signalling mechanisms	Transcription factors such as MYB (Myeloblastosis), bZIP (basic Leucine Zipper), and WRKY (defined by the conserved WRKYGQK motif) families are involved in metabolic regulation in model species [119,120].	The regulatory hierarchy linking carbon and nitrogen metabolism to secondary metabolite biosynthesis and stress signalling is not yet established in moringa.	Identify and characterise key genes, transcriptional regulators and hormonal cues (e.g., abscisic acid, jasmonic and salicylic acid) that govern metabolic reconstitution during cold stress adaptation.
Stress specific metabolite dynamics	Individual stresses modulate the accumulation of compatible solutes and antioxidants such as proline and phenolics [92,120].	Comparative profiling of metabolite changes under combined or sequential stresses is lacking, this limits the understanding of metabolite plasticity.	Conduct time and multi stress metabolomic analyses to identify synergistic or antagonistic interactions between metabolic responses.
Tissue specific metabolite responses	Most existing studies emphasise moringa leaf metabolites under stress [3,42,63].	The metabolic functions of roots and other organs, particularly their role in synthesis and systemic signaling, remain underexplored.	Implement tissue-specific metabolite mapping and transcriptomic profiling to reveal organ-dependent metabolic adjustments under stress environment.
Functional validation of metabolites	Correlations between metabolite accumulation and stress tolerance have been reported to be involved in metabolic regulation in model species [119,120].	Functional validation of key metabolites or genes through biochemical assays or genetic manipulation remain undefined in moringa.	Utilise functional genomics, enzyme assays, and heterologous expression systems to validate the roles of candidate metabolites in stress tolerance.
Ecological and allelopathic integration	Several secondary metabolites in moringa exhibit allelopathic and ecological relevance [121].	The ecological significance of stress-induced metabolites in mediating interspecific interactions or rhizosphere ecology remains poorly understood.	Examine how stress-driven metabolic shifts influence allelopathic potential and rhizosphere interactions to link physiology with ecological function.

## Data Availability

No new data were created or analyzed in this study.
